# Concerns about the interpretation of subgroup analysis. Reply.

**DOI:** 10.1172/JCI156711

**Published:** 2022-01-18

**Authors:** Shilong Li, Pei Wang, Li Li

**Affiliations:** 1 Sema4, Stamford, Connecticut, USA.; 2 Department of Genetics and Genomic Sciences, The Icahn Institute for Genomics and Multiscale Biology, Icahn School of Medicine at Mount Sinai, New York, New York, USA.

**Keywords:** COVID-19, Epidemiology, Medical statistics

## The authors reply:

We appreciate Albuquerque et al.’s interest in our paper (1, 2), about which the authors of the Letter raised the concern that we did not accurately interpret the interaction test. Their Letter noted that “one should directly compare the estimates (interaction test)” and “the authors concluded that the association was only present in the African American population, which is not compatible with their analysis.”

We would like to clarify that our primary clinical question was whether use of ACE inhibitors (ACE-Is) and angiotensin receptor blockers (ARBs) is associated with the COVID-19 outcomes in each subgroup. We used a stratified analysis to answer the question, because when race/ethnicity serves as a nonspecific proxy for numerous (confounding) factors, these can be (partially) controlled for through stratification (3). Joint modeling of multiple groups is often used to gain power, but one needs to assume certain coherent distributions across different groups, which is not always true. Additionally, testing the interaction term is to assess association heterogeneity between groups; it does not directly address whether the treatment is effective in each group.

Specifically, we would like to elaborate on two points. First, our conclusion that the use of ARB was associated with a significant reduction in in-hospital mortality among African American patients but not non–African American patients was based on results from the stratified analysis. We reported that ARB in-hospital use was associated with reduced mortality in the African American stratum (OR = 0.196; 95% CI 0.074–0.516; *P* = 0.001) with statistical significance. On the other hand, the association in the non–African American stratum is not statistically significant (OR = 0.687; 95% CI 0.427–1.106; *P* = 0.122). As stated previously, our primary objective was to assess whether ACE-I/ARB use among African American patients is associated with COVID-19 mortality, rather than whether there is a difference between African American and non–African American patients. We were also aware that the estimated ORs across different stratum were not comparable as noted in (4–6).

Second, we performed the joint modeling of African American and non–African American patients as suggested by Knol and VanderWeele (6). In our study, ARB in-hospital use was associated with reduced mortality in the entire study population (OR = 0.560; 95% CI 0.371–0.846; *P* = 0.006). The interaction term added to the model was not significant (95% CI 0.185–1.292; *P* = 0.149). Interpreting interaction terms in logistic regression is complex and a significant interaction term in log-odds may not be significant in difference-in-differences for probability (7). Furthermore, the assumption of the additive effects and imbalanced sample sizes could impact the inference.

We believe these results and the interpretation are appropriate. We acknowledge that there are cases where comparing the interaction term in greater detail would be an important next step for understanding the association between COVID-19 mortality and race and ethnicity.

## Version 1 12/02/2021

In-Press Preview

## Version 2 01/18/2022

Electronic publication
